# 

**Published:** 1844-10

**Authors:** 


### THE
### WESTERN JOURNAL
### OF
### MEDICINE AND SURGERY:

EDITED BY

DANIEL DRAKE, M. D.

AND

LUNSFORD P. YANDELL, M. D.

PROFESSORS IN THE LOUISVILLE MEDICAL INSTITUTE,

AND

THOMAS W. COLESCOTT, M. D.

NO. LVIII.—OCTOBER, 1844.

NEW SERIES.

VOL. II.—NO. IV.

LOUISVILLE, KY.

PUBLISHED MONTHLY, BY PRENTICE AND WEISSINGER,

PROPRIETORS AND PRINTERS.

1844.

*This Number contains 4 sheets—Postage under* 100 *miles* 6 *cents, over* 100 *miles* 10 *cents.*

